# Prediction Models for Adverse Pregnancy Outcomes in India: Methodological Considerations for an Emerging Topic

**DOI:** 10.1007/s13224-021-01617-4

**Published:** 2022-03-10

**Authors:** Gavin Pereira

**Affiliations:** 1 Curtin School of Population Health, Curtin University, Perth, WA 6102 Australia; 2 enAble Institute, Curtin University, Perth, WA Australia; 3 Centre for Fertility and Health (CeFH), Norwegian Institute of Public Health, Oslo, Norway

**Keywords:** Stillbirth, Prediction models, Validation

## Abstract

Stillbirth is over-represented in lower and lower-middle-income countries and understandably this has motivated greater research investment in the development of prediction models. Prediction is particularly challenging for pregnancy outcomes because only part of the population is represented in observational research. Notably, unrecognised pregnancies and miscarriages are typically excluded from the development of prediction models and the consequences of such selection are not well understood. Other methodological challenges in developing stillbirth prediction models are within the control of the researcher. Identifying whether the intended model is for aetiological explanation versus prediction, attainment of a sufficiently large representative sample, and internal and external validation are among such methodological considerations. These considerations are discussed in relation to a recently published study on prediction of stillbirth after 28 weeks of pregnancy for women with hypertensive disorders of pregnancy in India. The predictive ability of this model amounts to the flip of a coin. Future screening based on such a model may be expensive, increase psychological distress among patients and introduce additional iatrogenic perinatal morbidities from over-treatment. Future research should address the methodological considerations described in this article.

## Introduction

Stillbirth remains a relatively neglected outcome in all countries. Accurate prediction of stillbirth has remained elusive, particularly in lower and lower-middle-income countries which account for at least 84% of all stillbirths globally [1]. If stillbirth can be predicted in such countries, a large number of cases might be prevented. In response to this significant global issue, Kumar et al. [2] developed a model for the prediction of stillbirth after 28 weeks of pregnancy for women with hypertensive disorders of pregnancy. Abruption, gestational hypertension, family history of hypertension, maternal education, low number of antenatal visits (< 4 visits) and low foetal weight (< 2000 g) were used as predictors. The authors reported one of the strongest model performance results that have been reported of any study to date: 80% sensitivity, 70% specificity and 85% AUC. These seemingly strong results may lead clinicians to adopt the authors’ risk calculator (Excel spreadsheet) as a screening tool for stillbirth. However, I caution against application of the risk calculator on the basis that the size of the study, the performance of the model and the applicability of the model have been overstated.

## Main Text

Although the study successfully motivated further endeavour for the development of such models in India, several design and reporting limitations hamper both inference and application. I propose the following methodological considerations for future studies on the topic based on our team’s experience with one of the largest studies (*n* > 5,500 stillbirths) on stillbirth prediction to date, published in Nature Scientific Reports [3]; externally validated studies on preterm birth [4, 5]; and a recent registered protocol [6].

Firstly, the research question should be well defined. A research question for prediction or prognosis [3] is different to the more commonly investigated goal of explanation (aetiology) [7] and warrants a targeted approach to study design, model development and bias assessment. Contrary to the implicit assumption by Kumar et al.[2], it is possible for single risk factors to be not statistically significantly associated with stillbirth yet be predictive when considered together with other risk factors. Internal validation via a single hold-out sample or n-fold cross-validation has its limitations but at least provides a degree of confidence in predictive performance because the validation set is somewhat independent of the data used to develop the model. Lack of internal validation, as is the situation in the study by Kumar et al.[2], motivates postponement of external validation until this is achieved, and consequently proscribes application of the model for screening. Model development for stillbirth prediction would typically require observation of large numbers of stillbirths of the order of thousands and perhaps tens of thousands (*cf.* 265 stillbirth cases used by Kumar et al.[2]); and acknowledgement that risk operates on a continuum and has uncertainty (*cf.* no point or interval estimates for predictions in the risk calculator by Kumar et al.[2]). Finally, the importance of specificity must be maintained at levels at or above, say 90–95% [3] (*cf.* 70% used by Kumar et al.[2]) to minimise unnecessary intervention for pregnancies that would otherwise end in a healthy live birth with minimal maternal and child morbidities. Based on the ROC curve, if Kumar et al.[2] reported sensitivity at 95% specificity (comparable with other studies), sensitivity would reduce to approximately 50%—the flip of a coin. There is real possibility that screening based on a sub-standard test will be expensive, increase psychological distress among patients and introduce additional iatrogenic perinatal morbidities from over-treatment.

## Discussion

It is commendable that the authors and the World Health Organization South-East Asian Region (WHO SEAR) invested in the development of a database for congenital anomalies (SEAR-NBBD) and that the database includes stillbirths. Ideally researchers would require a registry of all births to enable estimation of incidence (or perhaps more accurately, prevalence at birth) and to ensure the study sample is both representative of pregnancies that end in stillbirth as well as live birth. Consequently, it is unclear as to whether the performance for the model reported by Kumar et al. [2] is impacted by selection of the control population. Given the improvement in collection of health data by health facilities and governments, the increased attention towards personalised medicine and the burgeoning field of data science, it will not be surprising to see a rapid increase in the development of models for prediction of adverse pregnancy outcomes in India. Such early attempts to derive prediction models for stillbirth are a step in the right direction and address the paucity of such studies on the topic.

## Conclusion

The rate of progress towards the development of a highly performing useful prediction model will be greatly sped up by considering the methodological considerations described in this article.
